# Exenatide ameliorates hepatic steatosis and attenuates fat mass and *FTO* gene expression through PI3K signaling pathway in nonalcoholic fatty liver disease

**DOI:** 10.1590/1414-431X20187299

**Published:** 2018-06-14

**Authors:** Shan Li, Xiaoman Wang, Jielei Zhang, Jingyi Li, Xiaogang Liu, Yuanyuan Ma, Chao Han, Lixia Zhang, Lili Zheng

**Affiliations:** 1 Department of Endocrinology, the First Affiliated Hospital of Zhengzhou University, Zhengzhou, Henan, China; 2 Department of Breast Surgery, the First Affiliated Hospital of Zhengzhou University, Zhengzhou, Henan, China; 3 Department of Pharmacy, the First Affiliated Hospital of Zhengzhou University, Zhengzhou, Henan, China

**Keywords:** GLP-1, FTO, NAFLD, PI3K, Hepatic steatosis

## Abstract

Non-alcoholic fatty liver disease (NAFLD) is a common disease associated with metabolic syndrome and can lead to life-threatening complications like hepatic carcinoma and cirrhosis. Exenatide, a glucagon-like peptide-1 (GLP-1) receptor agonist antidiabetic drug, has the capacity to overcome insulin resistance and attenuate hepatic steatosis but the specific underlying mechanism is unclear. This study was designed to investigate the underlying molecular mechanisms of exenatide therapy on NAFLD. We used *in vivo* and *in vitro* techniques to investigate the protective effects of exenatide on fatty liver via fat mass and obesity associated gene (*FTO*) in a high-fat (HF) diet-induced NAFLD animal model and related cell culture model. Exenatide significantly decreased body weight, serum glucose, insulin, insulin resistance, serum free fatty acid, triglyceride, total cholesterol, low-density lipoprotein, aspartate aminotransferase, and alanine aminotransferase levels in HF-induced obese rabbits. Histological analysis showed that exenatide significantly reversed HF-induced lipid accumulation and inflammatory changes accompanied by decreased FTO mRNA and protein expression, which were abrogated by PI3K inhibitor LY294002. This study indicated that pharmacological interventions with GLP-1 may represent a promising therapeutic strategy for NAFLD.

## Introduction

Non-alcoholic fatty liver disease (NAFLD) is one of the most common liver diseases associated with obesity, insulin resistance, and type 2 diabetes mellitus (1). It encompasses a variety of histological conditions ranging from simple steatosis to non-alcoholic steatohepatitis, liver fibrosis, cirrhosis, and hepatocellular carcinoma, of which cirrhosis and hepatocellular carcinoma are the two life threatening complications of NAFLD (2). Previous studies have confirmed that hepatic steatosis mostly results from an increased free fatty acid (FFA) supply to the hepatocytes due to increased lipolysis and/or increased intake of dietary fat, which contributes to 60% of hepatic fat content (3). Therefore, fat accumulation in the liver is a key factor for the development of dyslipidemia and insulin resistance. Clinical studies have shown that weight loss can improve fatty liver, and that reduced liver fat content confers lower serum fasting insulin and triglyceride (TG) concentrations relative to subjects with high levels of liver fat (4). Weight reduction and adequate control of diabetes (main complication) is the standard treatment for NAFLD.

Glucagon-like peptide (GLP)-1, an incretin, is secreted from the L cells in the small intestine and has the capacity to regulate plasma glucose level by inducing islet β-cell insulin secretion and inhibiting glucagon secretion. In light of these properties, GLP-1 administration was recently approved for use in diabetic hyperglycemia. Other than its role in maintaining blood glucose level, GLP-1 is associated with an increase in insulin sensitivity of peripheral tissue, delay in gastric emptying, early satiety, and suppression of appetite (5). These additional actions of GLP-1 are associated with body weight reduction. Furthermore, earlier studies have suggested that GLP-1 has favorable action on hepatocytes; it increases sensitivity of hepatocytes to insulin and enhances lipid metabolism through oxidation and hydrolysis. GLP-1 receptors are widely distributed across different organs including skeletal muscles and adipocytes. Oxidation of fatty acids in the skeletal muscles and storage of triglycerides in adipocytes play an important role in determining entry of fatty acids into the liver. GLP-1 can modify lipid metabolism by affecting the two above-mentioned steps and thus can reduce influx of fatty acids into the hepatocytes. Recently, GLP-1R expression was identified in human hepatocytes and cultured rodent hepatocytes, and a GLP-1R agonist appeared to reduce hepatic lipid storage in ob/ob mice (6,7). In addition, 6-month treatment of type 2 diabetic patients with hepatic steatosis with a GLP-1 receptor agonist promoted noticeable weight loss and significant reduction of intrahepatic lipid levels (8,9). However, the half-life of circulating GLP-1 is just 2 min *in vivo*, due to its rapid identification and cleavage by dipeptidyl peptidase-IV (DPP-IV) (10). Exenatide is a GLP-1R agonist that is resistant to DPP-IV and has a longer half-life, which makes it attractive for clinical applications (10).

The fat mass and obesity-associated gene (*FTO*) was discovered in a genome-wide association study for obesity or obesity-related traits in 2007 (11). Some researchers reported that FTO expression is upregulated in NAFLD, where FTO may play an important role in modulating oxidative stress and NAFLD pathogenesis by increasing the level of lipid deposition in liver cells (12). PI3K pathway is known to regulate cellular growth, survival, and metabolism. The active form of PI3K is an oncogene, and mutation/overexpression of PI3K is associated with cancer (13).

Currently, no experimental data of exenatide on fatty liver patients are available. Therefore, we investigated whether the protective role of exenatide on NAFLD is mediated via *FTO* gene expression and through modulation of PI3K signaling pathway in high-fat (HF) diet-induced obese New Zealand rabbits and related cell culture models.

## Material and Methods

### Animal preparation

Twenty-four male New Zealand rabbits (8 weeks of age) were purchased from the Vince Animal Experiments Co. (China). All rabbits were maintained under standardized conditions (21°C, 41–62% humidity) with a regular 12-h light/dark cycle as well as free access to water and a laboratory diet. The animals were fed with normal chow diet (control, 10 kcal % fat, 20 kcal % protein, and 70 kcal % carbohydrate). The HF diet (HFD) (45 kcal % fat, 20 kcal % protein, and 35 kcal % carbohydrate), which was provided by Henan Medical Laboratory Animal Center, was used to induce obesity and fatty liver. The experimental diets were manufactured in accordance with AIN-93M. Each group ate roughly the same number of calories per day. After 12 weeks of the chow diet or the HFD challenge, rabbits were divided randomly into 3 groups (n=8/group) as follows: normal chow diet + saline (control group), HFD + saline (HF), and HFD + exenatide (HF-Ex) (12). Rabbits were treated with a daily subcutaneous injection of exenatide (24 nmol/kg; Byetta, ID: C038173, Eli Lilly and Company, USA) or normal saline for 8 weeks (9). Food intake and body weight were monitored twice weekly during the period of exenatide administration. By the end of the 20^th^ week, after 8-h fasting, all animals were sacrificed under anesthesia. Blood samples were obtained from the inferior vena cava and the liver tissues were removed, weighed, and immediately frozen in liquid nitrogen for storage at −80°C until use in subsequent analyses. All experiments were approved by the Zhengzhou University Animal Ethics Committees (ZZU-2016-123).

### Serum analysis

At the completion of the study, fasting blood samples were collected for analysis of glucose and insulin concentrations and homeostasis model of assessment (HOMA) (15). Additional sera were obtained to measure serum glucose, FFA, TG, total cholesterol (TC), low-density lipoproteins (LDL), aspartate aminotransferase (AST), and alanine aminotransferase (ALT). Serum samples were separated by centrifugation at 500 *g* for 10 min at 4°C and stored at −80°C until measurements were taken. Plasma concentrations were measured using an automatic biochemical analyzer (Roche Cobas 6000, Germany). Serum sample kits were purchased from Roche (Germany).

### Histological analysis

After sacrifice, liver tissues were removed from the animals and weighed. The hepatic index was obtained by dividing the wet liver weight by the rabbit weight and multiplying by 100. Macroscopic and microscopic analyses of specimens were performed in a blinded fashion. Liver tissues were fixed in 10% neutral buffered formalin and then embedded in paraffin. After deparaffinization and dehydration, 5 µm thick sections were stained with hematoxylin and eosin (H&E) using standard protocols. The tissues were histologically graded according to the NAFLD activity score system by two pathologists blinded to the treatment groups (14). Hepatic triglycerides were extracted from frozen tissue and measured by enzymatic assays (Sigma, USA). TG values were normalized to protein concentration.

### Superoxide dismutase (SOD) and malondialdehyde (MDA) levels

Prepared fresh liver tissue samples were ground in saline solution to make 10% liver tissue homogenates, followed by centrifugation at 500 *g* for 20 min at 4°C. The resulting supernatant was collected to measure the contents of SOD and MDA using specific kits according to the manufacturer's instructions. All commercial test kits were obtained from the Jiancheng Bioengineering Company (China).

### Cell culture

L02 cell lines were purchased from the Institute of Liver Diseases (Huazhong University of Science and Technology, China), and cultured in 6-well plates in RPMI 1640 medium (Gibco, USA) containing 10% fetal bovine serum (Gibco) with 1% penicillin/streptomycin (Gibco). Upon reaching 70–80% confluence, cells were pretreated with or without LY294002 at a final concentration of 50 μM (BiYunTian, China), a classic inhibitor of PI3K/Akt, in control or high-fat RPMI 1640 by adding 0.4 mM palmitic acid (Gibco) for 24 h. Afterwards, exenatide with a final concentration of 10 nM was added to the wells with control or HF medium for another 24 h. Each group was repeated in 4 wells.

### Oil Red O Staining

Accumulation of triglyceride content in the treated L02 cells was visualized by Oil Red O (Sigma-Aldrich) staining. Lipid accumulation was photographed with a BX51WI microscope (Olympus, Japan).

### Biochemical measurements

After incubation for 48 h, cells were lysed with 1% Triton X-100, and an automatic biochemical analyzer (Roche Cobas 6000, Germany) was used to detect ALT, AST, lactate dehydrogenase (LDH), and alkaline phosphatase (ALP) (Roche, Germany) in the supernatant, as well as the intracellular TG content.

### Total RNA extraction and qRT-PCR

Total RNA was extracted using Trizol (China). cDNA was synthesized by reverse transcription of 0.5 µg template RNA, following the manufacturer's instructions. Primers for PCR, which were synthesized by the Sangon Corporation (China), were as follows: 5′-GAAGCACTGTGGAAGAAGATGGAGG-3′; 5′-TCAGCAGGTAATGTTCGGGCAAT-3′ for FTO (191 bp); and 5′-AACGGATTTGGTCGTATTG-3′; 5′-GGAAGATGGTGATGGGATT-3′ (208 bp) for GAPDH. Real-time PCR was performed with the Bio-Rad CFX96 qPCR system, and the data were analyzed using the 2^ΔΔCT^ method (15).

### Western blot analysis

Protein was isolated with mammalian protein extraction reagent (CWBIO, Beijing, China). Protein concentrations were determined by Bradford protein assay kit (CWBIO, China). Aliquots of samples (50 μg protein) were separated by SDS-PAGE, blotted onto polyvinylidene fluoride (PVDF) membranes and the blots were blocked in 5% milk for 2 h. Antibodies included anti-FTO, phosphorylated (p)-AKT, AKT (1:1000; CST, USA). GAPDH (1:500; USA) served as a loading control. The blots were incubated first with antibodies at 4°C overnight, and then incubated with horseradish peroxidase-conjugated secondary antibody (1:5000) at room temperature for 1 h. The membranes were imaged with the Odyssey infrared imaging system (LI-COR Biosciences, USA). Band intensities were quantified by densitometry.

### Statistical analysis

Statistical analysis was done using SPSS software package 17.0 (SPSS Inc., USA) to perform one-way analysis of variance (ANOVA) and paired *t*-test, followed by Duncan's multiple range test. The results for each group are reported as means±SD, and P<0.05 was considered significant.

## Results

### Effect of exenatide on body weight, food intake, and liver index

**Figure 1:**
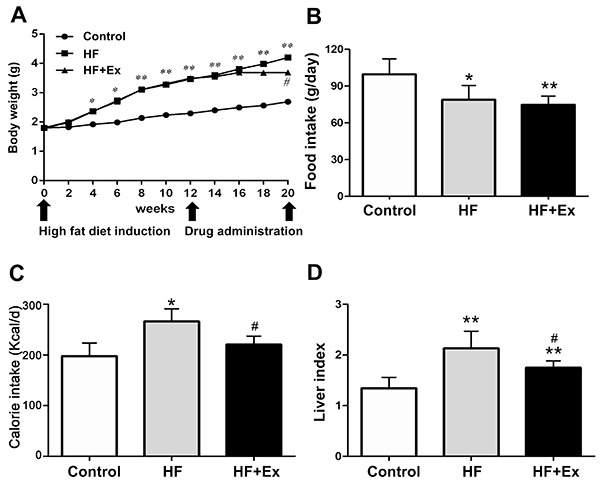
Changes in body weight, food intake, and liver index in exenatide (Ex)-treated high fat (HF)-induced obese rabbits (n=8). *A*, Body weight during HF diet feeding detected every 2 weeks. *B*, Average food intake during HF diet feeding. *C*, Average energy intake during the experimental period. *D*, Upon sacrifice at the end of 20 weeks, the liver index, which is equal to the liver weight/body weight x 100, was calculated. Data are reported as means±SD. *P<0.05, **P<0.01 compared with the control group; ^#^P<0.05 compared with the HF group (ANOVA).

Body weight of the rabbits was measured regularly over a period of 20 weeks. HF group body weight was higher than that of the control group from week 4, while body weight in the exenatide-treated group was significantly decreased compared to the HF group at week 20 (Figure 1A). During the experimental period, the food intake of the HF group was consistently and significantly decreased compared with the control group (P<0.01), while there was no difference between the exenatide-treated group and the HF group (Figure 1B). However, during the experimental period, the energy intake of the HF group was consistently and significantly increased compared with the control group (P<0.01, Figure 1C), while energy intake in the exenatide-treated group was significantly decreased compared to the HF group (P<0.01, Figure 1C). Exenatide was administered for the last 8 weeks in the HF-Ex group, whereas the control group and the HF group received saline during the last 8 weeks. The liver index of the HF group was higher than that of the control group (P<0.05), and the exenatide-treated group was significantly lower compared with the HF group (P<0.05, Figure 1D).

### Effect of exenatide on biochemical parameters

**Figure 2:**
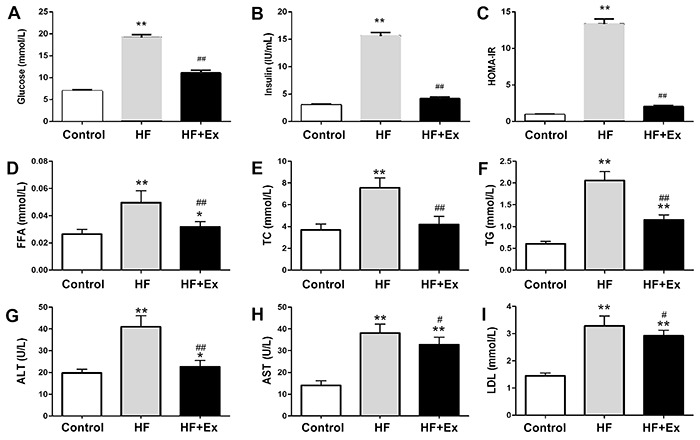
Effects of exenatide (Ex) on biochemical parameters of non-alcoholic fatty liver disease rabbits (n=8). At the end of the 8-week treatment, blood samples were collected for analysis of glucose (*A*), insulin (*B*), homeostasis model of assessment of insulin resistance (HOMA-IR) (*C*), free fatty acid (FFA) (*D*), total cholesterol (TC) (*E*), triglyceride (TG) (*F*), alanine aminotransferase (ALT) (*G*), aspartate aminotransferase (AST) (*H*), and low-density lipoprotein (LDL) (*I*) levels after overnight starvation. Data are reported as means±SD. *P<0.05, **P<0.01 compared with the control group; ^#^P<0.05, ^# #^P<0.01 compared with the high fat (HF) group (ANOVA).

After the end of the study period, blood samples were collected and evaluated. Serum glucose, insulin level, and degree of insulin resistance (IR) revealed significantly higher levels in the HF group compared to the control group, whereas the rabbits of the HF-Ex group had significantly lower levels of glucose, insulin, and IR compared to the HF group (Figure 2A and C). Exenatide also decreased the serum FFA, TC, and TG levels compared with the HF group (Figure 2D and F). In addition, the serum ALT, AST, and LDL levels in the HF-Ex group were also decreased compared to the HF group (Figure 2G and I).

### Effect of exenatide on liver fat accumulation

**Figure 3:**
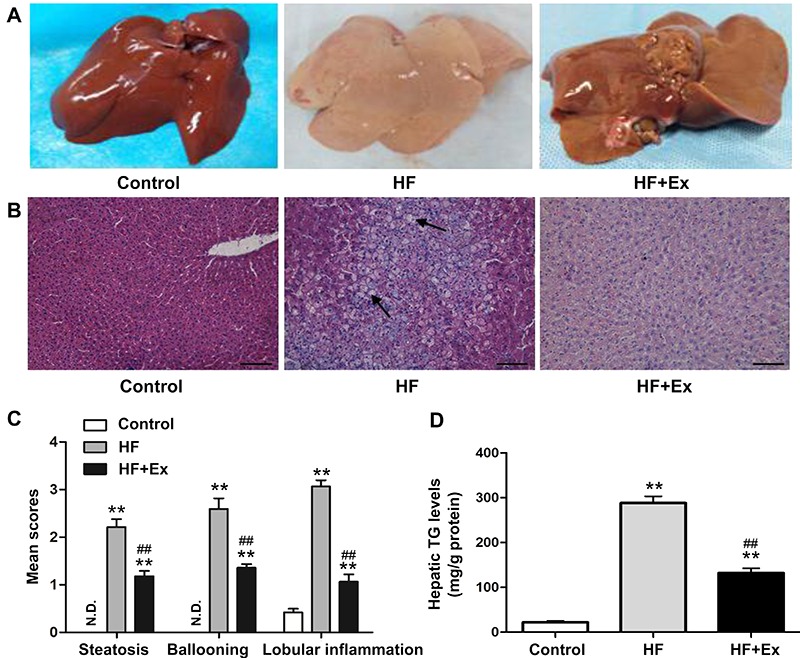
Histopathology of each group. *A*, Liver specimens from rabbits in each group are shown. *B*, Liver sections with hematoxylin and eosin (H&E) staining. Arrows indicate lipid droplets. The original magnification was ×200. Scale bars=500 μm. *C*, Non-alcoholic fatty liver disease activity scores were evaluated semi-quantitatively: steatosis (0–3), lobular inflammation (0–2), and hepatocellular ballooning (0–2). N.D., not detected. *D*, Hepatic triglycerides (TG) were extracted from frozen tissue and measured by enzymatic assays. Values of TG were normalized relative to protein concentration. Data are reported as means±SD (n=8). **P<0.01 compared with the control group; ^# #^P<0.01 compared with the high fat (HF) group (ANOVA).

Exenatide treatment significantly attenuated fat infiltration in rabbit livers (Figure 3A and B). Fresh liver tissue was bright red with smooth surfaces and sharp edges in the control group, but in the HF group, the liver tissue was yellow and swollen, with granular surfaces and blunt edges (Figure 3A). H&E staining showed that hepatic accumulation of lipids was significantly higher in the HF group compared with the control group and decreased with exenatide treatment (Figure 3B). In addition, mean scores for steatosis, ballooning, and lobular inflammation based on H&E staining was significantly decreased in the exenatide-treated group compared with the HF group (Figure 3C). In the HF group, hepatic TG levels were dramatically increased relative to the control group, whereas they were decreased in the exenatide-treated group (Figure 3D).

### Effect of exenatide on HF-induced liver oxidative stress

**Figure 4:**
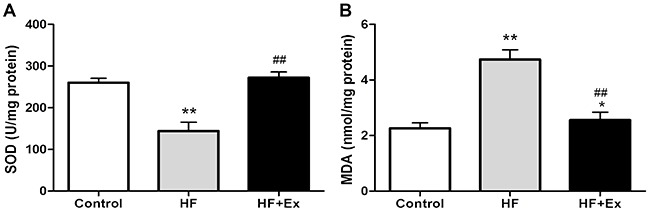
Effects of exenatide (Ex) on high fat (HF)-induced liver oxidative stress (n=8). After the 20-week study period, the contents of *A*, superoxide dismutase (SOD) and *B*, malondialdehyde (MDA) in livers from rabbits in each group were estimated. Data are reported as means±SD. *P<0.05, **P<0.01 compared with the control group; ^# #^P<0.01 compared with the HF group (ANOVA).

As shown in Figure 4A and B, after the end of study period, SOD levels were decreased and MDA levels were significantly increased in the HF group. However, SOD levels were increased and MDA levels were decreased in the exenatide-treated group compared with the HF group (Figure 4A and B).

### Effect of exenatide on FTO and pAKT expression in rabbit liver tissue

**Figure 5:**
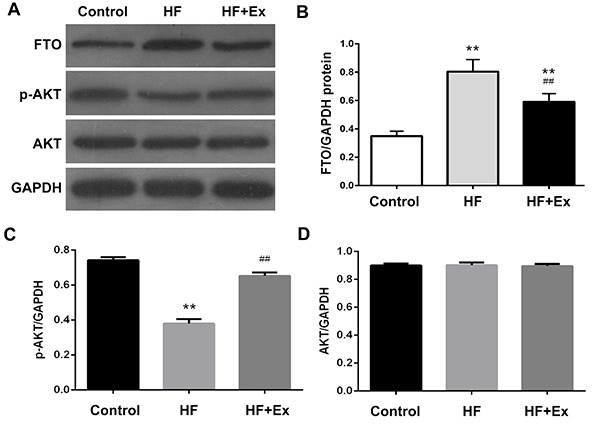
Effect of exenatide (Ex) on high fat (HF)-induced mass and obesity associated gene (*FTO*) expression in rabbit liver tissue (n=8). *A* and *B*, FTO protein expression; *C* and *D*, pAKT and AKT protein expression. Data were normalized relative to the GAPDH level for each sample and are reported as means±SD. **P<0.01 compared with the control group; ^# #^P<0.01 compared with the HF group (one-way ANOVA and paired *t*-test).

The results showed that protein level of FTO was significantly upregulated in the HF group compared with the control group, which was reversed by exenatide (Figure 5A and B). The level of pAKT was highest in the control and significantly lower in the HF group; however, the decreased level of pAKT was significantly increased following exenatide therapy (Figure 5A and C). The AKT levels were similar among the three groups (Figure 5D).

### LY294002 attenuated effects of exenatide on HF-induced lipid accumulation, liver enzyme, and TG levels in L02 cells

**Figure 6:**
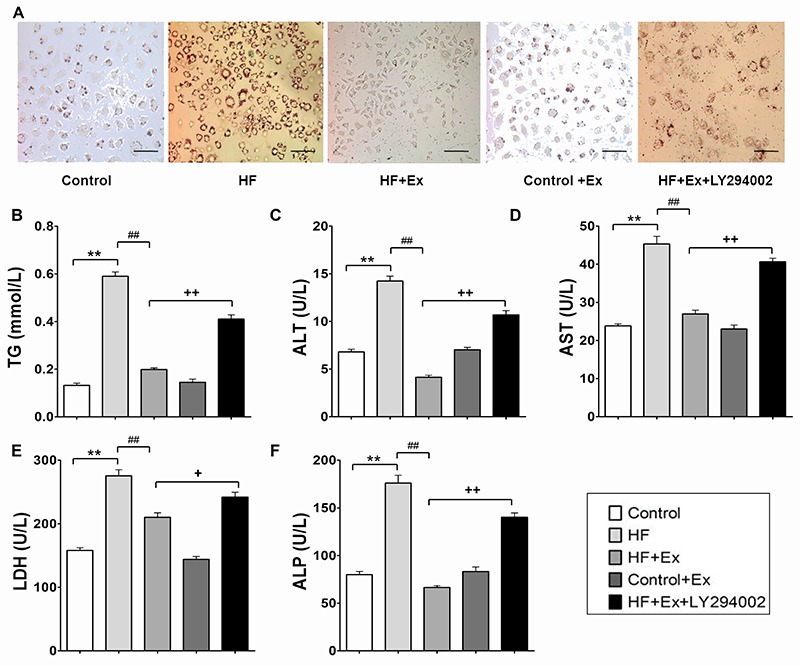
LY294002 attenuated effects of exenatide (Ex) on palmitic acid-induced lipid accumulation, liver enzyme, and triglyceride (TG) levels in L02 cells. *A*, Lipid accumulation in L02 cells was observed by Oil Red O staining. L02 cells stained with Oil Red O were examined by light microscopy (magnification ×400). Scale bars=500 μm. *B*–*F*, Serum contents of TG, alanine aminotransferase (ALT), aspartate aminotransferase (AST), lactate dehydrogenase (LDH), and alkaline phosphatase (ALP) were detected. Data are reported as means±SD (n=6). **P<0.01 compared with the control group; ^# #^P<0.01 compared with the high fat (HF) group; ^+^P<0.05, ^++^P<0.01 compared with the HF+Ex group (paired *t*-test).

Significantly increased lipid droplets were seen in the HF-treated cells compared to those in the control group, and exenatide decreased lipid accumulation. Meanwhile, LY294002 partially reversed the effect of exenatide in cells treated with HF medium (Figure 6A). We next examined liver enzyme and TG levels after exenatide exposure pretreated with or without LY294002. As Figure 6 B–F show, significant increases in TG, ALT, AST, LDH, and ALP concentrations were observed in HF-treated cells compared with the control group. Exenatide decreased TG, ALT, AST, LDH, and ALP concentrations in cells treated with HF, and LY294002 reversed the effects of exenatide.

### LY294002 partially reversed exenatide-dependent inhibition of HF-induced *FTO* gene expression in L02 cells

**Figure 7:**
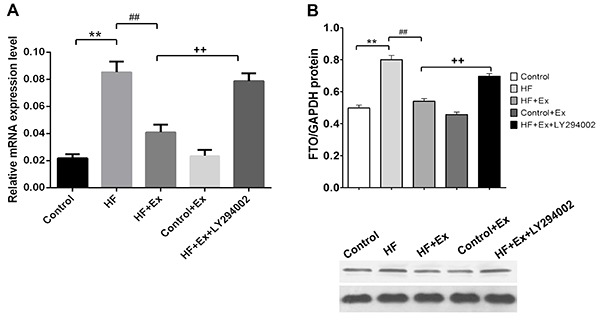
PI3K inhibitor LY294002 partially reversed exenatide (Ex)-dependent inhibition of palmitic acid-induced mass and obesity associated (*FTO*) gene expression in L02 cells. Total RNA and protein were extracted from L02 cells treated in each group and used to assess *A*, FTO mRNA expression and *B*, FTO protein expression by qRT-PCR and western blot, respectively. Data were normalized relative to the GAPDH level for each sample and are reported as means±SD (n=6). **P<0.01 compared with the control group; ^# #^P<0.01 compared with the high fat (HF) group; ^++^P<0.01 compared with the HF+Ex group (paired *t*-test).

As shown in Figure 7A and B, there was a significant increase in both mRNA expression and protein levels of FTO in cells treated with HF compared with control cells. Exenatide decreased FTO mRNA expression and protein levels in cells treated with HF, and LY294002 reversed the effects induced by exenatide.

## Discussion

NAFLD is an emerging public health concern, and this condition occurs even more frequently in patients with metabolic syndrome. The prevailing theory for the pathogenesis of NAFLD is closely associated with lipid accumulation within hepatocytes and lipotoxicity caused by oxidative stress from increased lipid peroxidation, mitochondrial dysfunction, and high ROS production.

Currently, no effective therapy for NAFLD has been established. One of the most promising approaches to optimize elimination of liver fat is the use of agents that improve insulin sensitivity. GLP-1 is a new drug approved for the treatment of diabetic hyperglycemia that improves insulin resistance by acting as an incretin. Xu et al. reported that SIRT1 is crucial in mediating the effect of the GLP1 receptor agonist exenatide on ameliorating hepatic steatosis by down-regulating lipogenic-related protein, including SREBP-1c and PNPLA3 (9). Zheng et al. reported that the SIRT1/heat shock factor 1/HSP pathway is essential for exenatide-alleviated, lipid-induced ER stress and hepatic steatosis (16). To further address the effects and mechanism, here we investigated the effects of exenatide on hepatic fat accumulation and inflammation and PI3K signaling pathway in the liver.

The results of this study demonstrated that treatment with the GLP-1 agonist exenatide resulted in a reduction in body weight and improvement in fat accumulation in liver tissues in HF-induced NAFLD animal model. Other findings showed that exenatide lowered glucose, insulin, and insulin resistance, which was consistent with a previous study of GLP-1 analogs in patients of type 2 diabetes (17). We found that exenatide limited HF-induced weight gain and reduced energy intake, which was consistent with a previous study of a reduction in both body weight and appetite in response to exposure of another GLP-1 agonist, exendin-4 (18). However, although GLP-1 receptor signaling accelerates plasma clearance of triacylglycerol and glucose by activating brown adipose tissue in mice (19), an increase in brown adipose tissue may be an additional mechanism whereby pharmacological GLP-1R activation controls energy balance (20).

Recent studies showed that the direct effect of GLP-1 on hepatocytes from human subjects with non-alcoholic steatohepatitis could be mediated by activating genes involved in insulin sensitivity and fatty acid oxidation, while weight loss could decrease hepatic steatosis while increasing insulin sensitivity (21,22). Wang and coworkers also reported that exenatide reduces hepatic cells and mitochondrial structural anomaly and improves insulin resistance in concert with improvements in insulin sensitivity and mitochondrial function activation, concomitantly with reductions in oxidative stress (23). In the present study, we verified that exenatide reduced oxidative stress by increasing SOD and decreasing MDA.

*FTO*, which was first discovered by the Frayling group (11), served as the first reliable obesity-related candidate gene; this gene is associated with obesity and type 2 diabetes. From these results, they concluded that the *FTO* gene could be functionally involved in energy homeostasis and, especially, in the control of energy expenditure. In our study, histological images of HF rabbit liver tissue showed a significant increase in lipid droplets in the liver and that exenatide treatment inhibited the development of NAFLD as indicated by changes in HF-induced plasma FFA levels and occurrence of hepatic steatosis in obese rabbits. A study showed that *FTO* gene expression was upregulated in the liver of NAFLD rats and that *FTO* overexpression enhanced oxidative stress and increased lipogenesis in hepatocytes, which are related to NAFLD pathology (24). The present study found that HF indeed significantly increased the expression of the *FTO* gene, and these changes were accompanied by variations in biomarker MDA and SOD for oxidative stress. Furthermore, exenatide reduced HF-induced *FTO* gene levels and reversed the changes in MDA and SOD in the NAFLD animal model, which in turn inhibited the development of NAFLD.

The PI3K/Akt signaling pathway, which is an insulin downstream molecular pathway closely associated with the development of insulin resistance, plays a vital role in various biological processes such as glucose transport, cell cycle regulation, cell metabolism, cell growth, and apoptosis. Previous study results showed that levels of PI3K and Akt proteins in NAFLD rat liver were significantly decreased compared to the control groups (25). Li and coworkers (26) found that Akt could regulate the process of hepatic glucose and lipid metabolism by suppressing fatty acid oxidation gene expression. GLP-1R activation has been shown *in vitro* to promote peripheral cell/tissue differentiation and proliferation by engaging PI3K/Akt, as well as to prevent apoptosis via a PKA/PI3K/Akt-dependent pathway (27,28). In the present study, we found that the effects of exenatide were associated with activation of p-Akt, which may suppress fatty acid oxidation gene and promote hepatocyte regeneration, thus inhibiting the development of NAFLD.

Based on the above studies, we concluded that the protective effects of GLP-1-reversing hepatic steatosis are dependent on inhibition of FTO gene expression via the PI3K signaling pathway, resulting in subsequent changes in oxidative stress biomarkers. These findings indicate that pharmacological intervention targeting to GLP-1 may be promising for the prevention and treatment of NAFLD.
